# Pseudoinfarction pattern in a patient with hyperkalemia, diabetic ketoacidosis and normal coronary vessels: a case report

**DOI:** 10.1186/1752-1947-4-115

**Published:** 2010-04-26

**Authors:** Antonios Ziakas, Christos Basagiannis, Ioannis Stiliadis

**Affiliations:** 1 First Department of Cardiology, AHEPA University Hospital, Saint Kiriakidi Street, 54636, Thessaloniki, Greece

## Abstract

**Introduction:**

A rare electrocardiographic finding of hyperkalemia is ST segment elevation or the so called 'pseudoinfarction' pattern. It has been suggested that hyperkalemia causes the 'pseudoinfarction' pattern not only through its direct myocardial effects, but also through other mechanisms, such as anoxia, acidosis, and coronary artery spasm.

**Case presentation:**

A 33-year-old Caucasian woman with insulin-treated diabetes presented with continuous epigastric pain of four hours duration. Her coronary heart disease risk factors apart from diabetes included hypercholesterolemia and smoking. Her initial electrocardiogram revealed ST segment elevation in the anteroseptal leads consistent with anterior myocardial infarction. Blood tests revealed hyperglycemia, hyperkalemia, metabolic acidosis and urine ketones, while a bed-side cardiac echocardiogram showed no segmental wall motion abnormality. We provisionally diagnosed diabetic ketoacidosis that was possibly precipitated by acute myocardial infarction, as there were findings in favor of (epigastric pain, electrocardiogram pattern, presence of 3 coronary heart disease risk factors) and against (young age, normal echocardiogram) the diagnosis of acute myocardial infarction. We performed cardiac angiography in order to exclude an anterior acute myocardial infarction, which could lead to myocardial damage and possible severe complications should there be a delay in treatment. Angiography revealed normal coronary arteries. During the procedure, ST segment elevation in the anteroseptal leads was still present in our patient's electrocardiogram results.

**Conclusion:**

ST segment elevation is a rare manifestation of hyperkalemia. In our patient, coronary spasm did not contribute to such an electrocardiography finding.

## Introduction

It has been reported that hyperkalemia can rarely produce abnormal ST segment elevation simulating an acute myocardial infarction [1-7]. This electrolyte abnormality influences the electrocardiogram (ECG) not only through its direct myocardial effects, but also through other yet vaguely understood mechanisms, such as anoxia, acidosis, and perhaps impaired contractility [1,2]. We present the case of a patient with diabetic ketoacidosis and hyperkalemia whose initial ECG showed a pseudoinfarction pattern, but an urgent coronary angiogram revealed normal coronary arteries.

## Case presentation

A 33-year-old Caucasian Greek woman presented to the emergency department of the Hospital with a continuous epigastric pain of four hours duration and intermittent vomiting. Her medical history included hypercholesterolemia and type 1 diabetes for 16 years treated with insulin injections twice daily. Our patient had omitted all insulin injections since 36 hours prior to presentation. Regarding coronary risk factors, apart from diabetes and hypercholesterolemia, she was a smoker of more than two packs of cigarettes daily.

**Figure 1:**
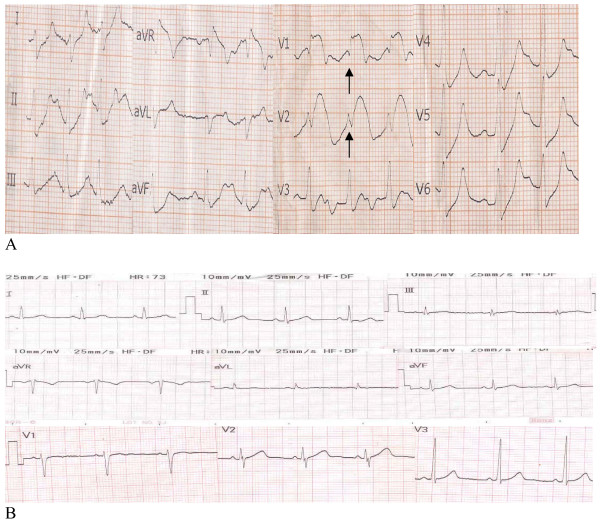
**(A) 12-lead electrocardiogram of our patient on admission showing ST segment elevation in the anteroseptal leads and intraventricular conduction delay**. (B) 12-lead electrocardiogram of our patient showing a complete resolution of the anteroseptal ST segment elevation and the intraventricular conduction delay.

On initial assessment she was drowsy with tachycardia (112 pulses/minute), tachypnoea (28 breaths/minute) and hypotension (85/44 mmHg). A physical examination of her abdomen had normal results. Her initial ECG revealed sinus tachycardia, ST segment elevation in the anteroseptal leads consistent with anterior myocardial infarction, and intraventricular conduction delay (Figure 1A). A urine dipstick test detected ketones, bedside capillary testing using a glucometer showed high glucose concentrations, and arterial blood gas analysis indicated metabolic acidosis (pH = 7.16, carbon dioxide partial pressure = 13 mmHg, oxygen partial pressure = 123 mmHg, bicarbonate concentration = 4 mmol/L, base excess = -24 mmol/L). We provisionally diagnosed diabetic ketoacidosis, possibly precipitated by an acute myocardial infarction.

We initially treated our patient with fluid replacement with normal saline, intravenous insulin at seven units/hour, sodium bicarbonate, aspirin, clopidogrel, and low molecular weight heparin. Biochemical results showed the following serum concentrations: potassium = 7.2 mEq/L, sodium = 127 mEq/L, urea = 97 mg/dl, creatinine = 2.26 mg/dl, and glucose = 676 mg/dl. A bedside cardiac ECG showed no segmental wall motion abnormality and a normal ejection fraction. As there were findings both for (epigastric pain, ECG pattern, presence of three coronary heart disease risk factors) and against the diagnosis of acute myocardial infarction (young age, normal ECG), we performed coronary angiography in order to exclude anterior acute myocardial infarction, which could lead to severe myocardial damage and possible severe complications (heart failure, among others) if treatment was delayed. During angiography, which revealed normal coronary arteries, ST segment elevation in the anteroseptal leads was still present in her ECG findings.

A repeat biochemical test after three hours showed the following values: sodium = 130 mEq/L, potassium = 4.9 mEq/L, and glucose = 255 mg/dl. A repeat ECG showed a complete resolution of the anteroseptal ST segment elevation and intraventricular conduction delay (Figure 1B). Her troponin I concentration 12 hours after admission was normal (0.1 μg/L). Our patient subsequently made an uneventful recovery. When she was discharged seven days after, both her ECG and biochemical results were normal.

## Discussion

Our patient, who had known diabetic ketoacidosis and hyperkalaemia, had initial ECG findings suggestive of a myocardial infarction, but urgent coronary angiography revealed normal coronary arteries.

Although total body potassium concentrations may be considerably depleted in cases of diabetic ketoacidosis, plasma potassium concentrations at the time of presentation are usually normal or high. Acidosis, which causes potassium ions to leave the cells, as well as insulin deficiency and renal impairment, all contribute to hyperkalemia [8]. Potassium concentrations above 6.0 mmol/L have been reported in 22% to 32% cases at the time of presentation [9,10].

Hyperkalemia has profound effects on myocardial conduction and repolarization and hence on surface ECG. There is a peaking of the T waves and sometimes shortening of the QT interval. The ST segment may virtually disappear and become incorporated into the proximal limb of the T wave. P wave amplitude progressively diminishes and eventually disappears when serum potassium concentrations are above 7.5 mmol/L. This may lead to sinoventricular rhythm. Intraventricular conduction defect is manifested as widening of the QRS, which often resembles right bundle branch block with either a left anterior or a left posterior hemiblock. A sine wave pattern may occur in patients with end-stage hyperkalemia [11].

A rare manifestation of hyperkalemia is ST segment elevation or 'pseudoinfarction' [1-7]. Because this pattern disappears after treatment, the term 'dialyzable current of injury' has been considered appropriate. It is debatable whether ST elevation is a primary repolarization abnormality or an artifact caused by the merging of the terminal R portion of the QRS with the T wave. It is possible that this electrolyte abnormality influences the ECG not only through its direct myocardial effects, but also through other yet vaguely understood mechanisms, such as anoxia, acidosis, and perhaps impaired contractility [1,2]. It has also been suggested that in cases with hyperkalemia due to diabetic ketoacidosis, changes in ECG are also due to other metabolic abnormalities specific to diabetic ketoacidosis [7].

It is interesting to note that severe diabetic ketoacidosis might be associated with myocardial necrosis, which might be due to an atherothrombotic process superimposed on a preexisting coronary artery disease or coronary artery spasm [12]. Furthermore, coronary spasm triggered by hyperkalemia has been suggested as a contributor to these ECG changes [13]. In our case, our patient underwent urgent cardiac angiography as the diagnosis of acute myocardial infarction was not definite. This was in order to absolutely exclude an anterior acute myocardial infarction, which could lead to myocardial damage and possible severe complications. It is interesting to note that angiography showed normal coronary vessels, while the ECG had changes suggestive of anterior myocardial infarction. To the best of our knowledge, this is the first case in the literature involving pseudoinfarction pattern due to hyperkalemia, in which cardiac angiography was performed during the transient ECG changes.

## Conclusions

We conclude that ST segment elevation is a rare manifestation of hyperkalemia, and in our case coronary spasm did not contribute to this electrocardiography finding. However, as we only report one specific case, conclusions on the relationship between coronary spasm and hyperkalemia's pseudoinfarction pattern should not be made until further studies are done. With the current emphasis on reducing door-to-needle times for thrombolysis or primary Percutaneous Coronary Intervention (PCI) to curtail morbidity and mortality from coronary artery disease, it is worth remembering that metabolic abnormalities can sometimes alter electrocardiographic appearances. Starting thrombolysis or proceeding to urgent cardiac catheterization before metabolic abnormalities are corrected may thus expose our patient to unnecessary treatment, along with its attendant risks.

## Competing interests

The authors declare that they have no competing interests.

## Authors' contributions

AZ and IS analyzed and interpreted patient data and were involved in the management of the patient (medical treatment, coronary angiography). CB was a major contributor in writing the manuscript. All authors read and approved the final manuscript.

## Consent

Written informed consent was obtained from our patient for publication of this case report and any accompanying images. A copy of the written consent is available for review by the Editor-in-Chief of this journal.
